# Exploring the Pharmacokinetic Profile of Remifentanil in Mid-Trimester Gestations Undergoing Fetal Intervention Procedures

**DOI:** 10.3389/fphar.2017.00011

**Published:** 2017-01-24

**Authors:** Judith A. Smith, Roopali V. Donepudi, Pedro S. Argoti, Anita L. Giezentanner, Ranu Jain, Noemi Boring, Elisa Garcia, Kenneth J. Moise

**Affiliations:** ^1^Department of Obstetrics, Gynecology and Reproductive Medicine, UTHealth McGovern Medical SchoolHouston, TX, USA; ^2^Department of Pharmacy, Memorial Hermann Hospital-Texas Medical Centre (TMC)Houston, TX, USA; ^3^The Fetal Center, Children's Memorial Hermann HospitalHouston, TX, USA; ^4^Department of Anesthesiology, UTHealth McGovern Medical SchoolHouston, TX, USA

**Keywords:** fetal anesthesia, pregnancy pharmacokinetics, remifentanil, twin-twin transfusion syndrome

## Abstract

**Background:** Indications for surgery during pregnancy have increased. Specifically fetal interventions have increased from conditions that were considered lethal like twin-twin transfusion syndrome and severe fetal anemia to non-lethal conditions like myelomeningocele. The optimal anesthetic agent for *in utero* surgery is yet to be determined. Success of the procedure is often dictated by the efficacy of the anesthetic to immobilize the fetus without over-sedating mom. Remifentanil is used as preferred agent due to its short half-life however pharmacokinetics in pregnancy is unknown.

**Objective:** To determine the pharmacokinetic parameters of remifentanil in a mid-trimester pregnant patient population undergoing fetal intervention.

**Study Design:** A validated liquid chromatography assay with ultraviolet absorbance was employed to estimate maternal serum remifentanil levels. Blood samples were obtained at baseline and at selected time points: 5, 15, 30, 45, 60 min after the beginning of the remifentanil infusion and at 15, 30, and 60 min post end of infusion.

**Results:** Ten pregnant patients were enrolled in the study however only eight patients had sampling obtained at all time points. The mean gestational age was 22.2 (±2.7) weeks, maternal age was 27.8 (±5.1) years and body mass index was 29.6 (±6.3). After receiving a continuous infusion of remifentanil, mean total dose was 975.3 μg, C_min_ was 2.0 ng/mL and C_max_ was 8.4 ng/mL. A two-compartment model best described the plasma remifentanil data. Mean pharmacokinetic parameters were: volume of distribution (Vd_c_) = 124.6 L (16.2–530.8 L), maternal remifentanil total clearance (Cl_t_) = 170.7 L/h (17.7–486.9 L/h), and half-life (t_½_) = 0.6 h (0.2–0.9 h). The maternal remifentanil area under the curve (AUC) ranged from 2.7 to 21.7 μg/L^*^h. The mean alpha-acidic glycoprotein was 124.8 mg/dL (81.3–149.8).

**Conclusion:** The pharmacokinetic profile of remifentanil in pregnant women is similar to previously reported general population profiles. This data did provide potential rationale for the clinical observations why when remifentanil is dosed based on non-pregnant guidelines, it did not uniformly provide adequate fetal immobilization as per anecdotal perception of operating fetal surgeons. These findings are important for the development of further clinical studies to optimize dosing for surgery during pregnancy including the estimation of placental transfer and total fetal exposure.

## Introduction

Despite the increasing popularity of specific anesthetic agents used for fetal intervention procedures such as remifentanil, very little information about their pharmacological and toxicological profiles in the maternal-fetal population exist (Dershwitz et al., 1995). Limited information exists about the long-term effects of fetal exposure to anesthetics, and yet, based on limited animal and human studies, a great public concern has been raised about the potential long-term neurotoxic effects of anesthetic exposure in early life (Loepke and Soriano, 2008). Fetal life is a period of active brain development, with a hypothesized greater susceptibility to environmental and pharmacologic insults (Palanisamy, 2012). The indications for fetal surgery have increased in recent years, with an expected increase in the number of procedures to be performed in the future (Deprest et al., 2010). Given the significant maternal and fetal morbidity inherent to fetal surgery, these procedures are reserved for conditions that are either lethal or associated with significant long-term disability, such as twin-to-twin transfusion syndrome, congenital diaphragmatic hernia and myelomeningocele. Additionally, fetal exposure to anesthetic agents can occur at any time during pregnancy for a maternal complication requiring operative intervention such as acute appendicitis.

Remifentanil is a category C potent ultra-short acting synthetic opioid drug. It is FDA approved for sedation and analgesia in both adults and children. It is usually combined with other medications for induction and maintenance of general anesthesia and is typically administered intravenously as a bolus or as a continuous infusion. Remifentanil is metabolized via rapid hydrolysis by non-specific plasma and tissue esterases. The terminal half-life is 10–21 min (Westmoreland et al., 1993).

When compared with other anesthetic agents such as diazepam for fetal intervention procedures, remifentanil produces maternal sedation and analgesia as well as fetal immobilization in a more rapid and pronounced manner. This has the potential to decrease the total surgical time and subsequently the length of fetal exposure to anesthetic agents (Van De Velde et al., 2005). Evidence also shows that remifentanil may have neuro-protective and other cell protective properties (Fodale et al., 2008; Jeong et al., 2012). However, the optimal dose needed to provide adequate fetal sedation is not clearly studied. Anecdotal experience has observed that when dosing is based on non-pregnant studies, it unfortunately does not uniformly provide adequate fetal immobilization. This led to the current study to determine the pharmacokinetic profile of this drug in pregnancy. The primary objective of this study was to define the pharmacokinetic (PK) parameters of remifentanil in a pregnant patient population in mid-gestation. A secondary objective of the study was to evaluate the alpha acidic glycoprotein (AAG) levels on remifentanil pharmacokinetics. AAG is a serum “stress plasma protein” with a short 7 to 10 day half-life that readily binds to medications. Remifentanil is reported to have greater than 80% plasma protein binding (PPB), with over 75% specifically to AAG, however the impact of gestational stress, such as twin transfusion syndrome, on AAG levels has not been evaluated. This basic knowledge of the maternal pharmacokinetics of remifentanil and better understanding of the impact of gestational stress on AAG levels will open the doors for further toxicological and fetal (PK/PD) research.

## Materials and methods

### Patient and study design

Patient enrollment was approved by the institutional review board (IRB) of the University of Texas Health Science Center at Houston and Children's Memorial Hermann Hospital, Houston, TX. The population selected included patients undergoing laser photocoagulation of placental anastomoses for twin-to-twin transfusion syndrome (TTTS). Patients underwent standardized ultrasound evaluation and counseling in order to determine if they were candidates for fetal intervention for TTTS. Only after they had been deemed appropriate surgical candidates by the faculty of The Fetal Center were they offered enrollment and consented to participate in the study.

### Remifentanil infusion

The remifentanil infusion was initiated at 0.05–0.08 mcg·kg^−1^·min^−1^ just prior to surgical procedure upon the transfer of the patient to the operating room table. The infusion was initiated with a higher dose followed by continuous intravenous infusion adjusting the rate based upon the patient and the fetus's sedation level and physiological response. The initial infusion rate and any changes made by the anesthesiologist or surgeon in order to attain optimal operative conditions were documented on the data collection sheet.

**Figure 1:**
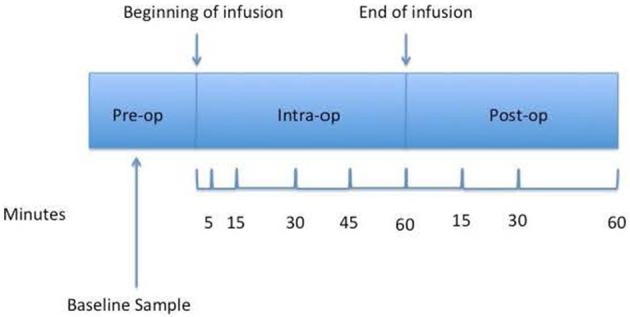
**Sample draw timeline**. The figure shows all time points at which blood samples were drawn.

An additional intravenous catheter was placed in the contralateral arm then the one used for the remifentanil infusion for pharmacokinetic (PK) sampling. Plasma concentrations of remifentanil were obtained at baseline and at selected time points: 5, 15, 30, 45, 60 min after the beginning of the remifentanil infusion and at 15, 30, and 60 min post end of infusion (Figure 1). Due to limitations set by the IRB for the number of samples that can be obtained from a pregnant patient, the above time points were chosen.

An initial baseline sample of 12 mL was drawn; 6 mL used for PK and the remaining 6 mL was used for determining the AAG levels. All the other samples were 6 mL. The intravenous catheter was flushed with normal saline after each blood sample collection to help prevent clotting thus, the initial 2 mL of blood was discarded prior to each PK sample being drawn. The samples were placed on ice, transported to the analytical core laboratory and processed within 15 min of collection. The collection tube was centrifuged under refrigeration (4°C) 1000 × G for 10 min to separate the cellular elements from the plasma. Maternal plasma samples were then stored at −80°C until the time of analysis.

### Remifentanil sample analysis

Remifentanil concentrations were quantified by validated high pressure liquid chromatography (HPLC) assay according to parameters described in the CDER Guidance for Industry Bioanalytical Assay Method Validation (Guidance for Industry: Bioanalytical Method Assay Validation, 2001). Remifentanil was isolated from maternal plasma samples by solid phase extraction after the addition of 10 μL of fentanyl 10 μg/mL as the internal standard. Liquid chromatographic separation was achieved by isocratic mobile phase: the mobile phase consisted of a composition of 60% of the following mixture: acetonitrile: methanol: 50 mM potassium phosphate buffer, pH3: water (180:120:48:652), and 40% methanol adjusted pH 7.2 with flow rate 1 mL/min with a elution on a Phenomenex PhenoSphere C1, 150 × 4.6 mm, 5 μm particle size packing analytical column (Phenomenex, Torrance, CA). The remifentanil peak eluted at 9.20 ± 0.2 min and fentanyl retention time was 11.50 ± 0.2 min identified by photodiode array detector at a wavelength of 210 nm. The remifentanil assay was found to be linear over 2–100 ng/mL with a correlation coefficient (*r*) of 0.997. The coefficient of variation for the remifentanil assay ranged from 0.16 to 14.9%. The alpha1-acidic glycoprotein (AAG) was determined using the Quantikine ELISA technique (R&D systems, Inc. Minneapolis, MN, USA).

### Pharmacokinetic analysis

The plasma concentration time data was fit using ADAPT II Software Version 4.0. (BMRS, University of Southern California, Los Angeles, CA, USA) to determine optimal PK model to describe the data (D'argenio and Schumitzky, 1979). Model discrimination was based on the Aikaike inspection criteria (AIC), Schwartz, weighted sum of squares, sum of squares and linear correlation (*R*^2^) as well as visual inspection of the data fit. Systemic exposure (AUC) was calculated: AUC (μg·L^−1^·h^−1^) = [total dose (μg)/total clearance (L/h)].

## Results

A total of 10 pregnant patients were enrolled in the study however only eight patients had complete sampling; these eight patients comprised the study population. The mean gestational age was 22.2 (±2.7) weeks. The mean maternal age was 27.8 (±5.1) years and BMI was 29.6 (±6.3). Five of the patients were Hispanic and 3 were Caucasian (Table 1). The mean C_min_ = 2.0 ng/mL and the mean C_max_ = 8.4 ng/mL after receiving continuous infusion of remifentanil with a mean total dose of 975.3 mcg. The mean remifentanil concentration per minute was 12.6 ± 7.8 ng/ml (Table 2).

**Table 1:** **Demographics and infusion data**.

**Patient**	**Age**	**Gestational age (weeks)**	**Race**	**Gravity and parity at the time of evaluation**	**TTTS stage**	**BMI**	**Starting infusion (mcg·kg^−1^·h^−1^)**	**Starting infusion dose (mcg/h)**	**Duration of infusion (min)**
1	28	21	White	G2P1	III	31	0.3	1480	85
2	30	26	White	G2P1	I	24	0.08	396	82
3	21	25.7	White	G2P1	IV	43	0.12	792	73
4	24	21.4	Hispanic	G4P3	I	21	0.2	672	81
5	32	18.9	White	G2P0	III	30	0.5	2280	84
6	33	21.4	Hispanic	G6P4	II	30	0.05	241	62
7	33	19.4	White	G3P1	II	29	0.1	366	67
8	21	23.6	Hispanic	G2P1	II	29	0.1	438	70

*Demographics and infusion data for each patient in the study*.

**Table 2:** **Pharmacokinetic results**.

	**Remifentanil C_min_ (ng/mL)**	**Remifentanil C_max_ (ng/mL)**	**Mean concentration during infusion (ng/mL)**	**Maternal total clearance Cl_t_ (L/h)**	**Volume of Distribution Vd_c_ (L)**	**Mean Half-life (h)**	**AUC (μg·L^−1^·h^−1^)**	**AAG level (mg/dL)**
Mean	2.0	8.4	4.8	170.7	124.6	0.6	10.6	124.8
Standard deviation	1.3	5.0	2.7	185.2	170.2	0.2	6.7	24.2
CV%	67.4	60.2	57.8	108.5	136.7	43.4	63.7	19.4

*Pharmacokinetic results with mean, standard deviation, and coefficient of variation (CV%)*.

A two-compartment model best described the plasma remifentanil data. Mean PK parameters were: volume of distribution (Vd_c_) = 124.6 L (16.2–530.8 L), maternal remifentanil total clearance (Cl_t_) was 170.7 L/h (17.7–486.9 L/h), and half-life (t_½_) was 0.6 h (0.2–0.9 h). The maternal remifentanil AUC ranged from 2.7 to 21.7 μg·L^−1^·h^−1^ (Table 2). The mean AAG was 124.8 mg/dL (81.3–149.8 mg/dL) in these pregnant patients.

## Discussion

The pharmacokinetics of remifentanil in normal healthy non-pregnant adults has been shown to be similar in both genders (Egan, 1995; Minto et al., 1997; Scott and Perry, 2005). The data from this study suggested that the PK profile in pregnant women is similar to other healthy non-pregnant women with a few notable differences. The mean volume of distribution in this study was 124.6 L (±170.2), which is higher than normal healthy individuals (25–40 L) (Westmoreland et al., 1993). This was approximately a 3.3-fold increase in the volume of distribution, which is not unexpected in pregnancy. There is a 40–50% increase in the maternal blood volume in pregnancy which starts early in the first trimester and peaks at around 32 weeks gestation (Hytten and Paintin, 1963). Thus, this increased maternal circulating volume contributes to the greater volume of distribution noted in the current study in pregnant patients. This might be an attributing factor to the clinical observation of delay in fetal immobilization that had prompted this study. Previous studies have shown a significant amount of transfer of remifentanil across the placenta (Welzing et al., 2011; Heesen et al., 2013). However, there is also rapid metabolism and redistribution of remifentanil Kan et al. (1998). have shown an umbilical vein: maternal serum (UV:MA) ratio of 0.88 ± 0.78 and an umbilical artery: umbilical vein ratio of 0.29 ± 0.07. This was similarly demonstrated in a randomized trial by Ngan Kee et al. (2006) where the UV:MA ratio was 0.73 ± 0.17. During the current PK study there was a concurrent pharmacokinetic study in pregnant women undergoing intrauterine transfusions that also received sedation with remifentanil that observed a similar transfer fraction (UV:MA) of 0.64 ± 0.52. These studies would indicate that transplacental transfer/distribution of remifentanil into the fetal compartment may also contribute to the greater volume of distribution noted in the current study.

The maternal remifentanil clearance in our study was 170.7 L/h (±185.2), which is lower than the clearance previously reported in non-pregnant women (250–300 L/h) (Westmoreland et al., 1993). The half-life in our study was 0.6 h (±0.2) as compared to non-pregnant women where it ranges from 0.2 to 0.4 h (Westmoreland et al., 1993). We therefore found a 1.8 fold decrease in the remifentanil clearance, which would explain the increase in the half-life by about 1.5-fold. Typically in pregnancy the renal blood flow and the glomerular filtration rate (GFR) are increased by approximately 50% (Dunlop, 1981). This increase in the GFR results in an increase in the elimination of medications that are cleared by renal excretion and a corresponding shorter half-lives (Pacheco and Hankins, 2013). However, remifentanil is predominantly metabolized by non-specific circulating esterases to an acid metabolite GI-90291 (Westmoreland et al., 1993). This metabolite is excreted through the kidney. The clearance of remifentanil is therefore not affected directly by the maternal renal function.

The decrease in clearance could be related to the increase in the AAG levels noted in the current study as compared to the non-pregnant population. The mean level of 125 mg/dL observed in these patients was nearly two-fold higher than the mean level of 62.6 mg/dL previously reported by Chu et al. (1981) Approximately 70% of remifentanil is bound to plasma proteins; one third to albumin but most of it, greater than two-thirds, is bound to AAG decreasing its free-fraction available for drug activity. Certain plasma proteins like albumin are decreased in pregnancy, while others like sex hormone binding globulins are increased (Pacheco and Hankins, 2013). These changes in the protein concentrations are important in determining the drug response (Wood and Wood, 1981). AAG is a physiological stress protein, hence in conditions associated with more physiological stress, higher levels of AAG will be observed. For example, Chu et al. (1981) did not find any difference in the AAG levels in uncomplicated pregnancies when compared to non-pregnant women (62.6 ± 18.8 mg/dL). However, they did find that pregnancies complicated by acute or chronic inflammation resulted in an increase in AAG levels (Chu et al., 1981). The current study found that the AAG was elevated (124.8 mg/dL ± 24.2). Although the study patients did not have any significant medical history, their pregnancies were complicated by the need for fetal surgery. The effects of fetal surgery on maternal stress have not been studied, however any surgery would appear to cause significant psychological stress. Coussons-Read et al. (2007) demonstrated an increase in the pro-inflammatory cytokines and acute phase reactants in cases of increased prenatal stress. The increased AAG concentration results in an increase in the amount of protein-bound of remifentanil, decreasing the free fraction of the active remifentanil. Hence the elevated AAG levels may also have contributed to the clinical observations of delay fetal immobilization when pregnant women are dosed based on non-pregnant dosing guidelines. In addition, subsequently a two-fold decrease in its clearance that was observed in this study. As a result of the decreased clearance and longer elimination half-life, a more prolonged interval may be necessary to reach a steady state in the pregnant woman. The delay in reaching steady state is another factor have contributed to the clinical observations of delay fetal immobilization when pregnant women are dosed based on non-pregnant dosing guidelines.

This study is limited by its small sample size. While enrolled 10 pregnant patients and were able to obtain adequate samples from 8 women. This is a prospective study of pregnant women in their second trimester undergoing a fetal procedure and we were able to obtain pharmacokinetic studies in these women, which have enhanced our understanding of remifentanil effects in pregnant women.

In conclusion, the PK profile of remifentanil in pregnant and non-pregnant women was similar but with differences in volume of distribution, clearance, half-life and AAG levels. While limited by a small sample size, this data did provide some potential rationale for the clinical observations why when remifentanil is dosed based on non-pregnant guidelines, it does not uniformly provide adequate fetal immobilization hence higher remifentanil doses may be required to achieve adequate fetal immobilization. Clinically these higher remifentanil doses may not have a significant impact on patient outcomes given the benefits of remifentanil in obtaining adequate fetal analgesia and its rapid clearance from the fetal circulation. These findings are important for the development of further clinical studies to optimize dosing for surgery during pregnancy including the estimation of placental transfer and potential total fetal exposure.

## Author contributions

JS: Co-Investigator, Study conception, design, sample analysis, pharmacokinetic analysis, manuscript preparation. RD: Patient consent, data analysis, manuscript preparation. PA: Co-Principal Investigator, study conception, design, patient consent, data analysis, manuscript preparation. AG: Co-Investigator, study design, Sample acquisition, data collection, patient monitoring, manuscript preparation. RJ: Sample acquisition, data collection, patient monitoring. NB and EG: Patient navigator, sample acquisition, data monitoring. KM: Principal Investigator, study conception, design, patient enrollment, patient consent, data analysis, manuscript preparation.

### Conflict of interest statement

The authors declare that the research was conducted in the absence of any commercial or financial relationships that could be construed as a potential conflict of interest.
